# Two new species of *Lysiteles* Simon, 1895 from Cibagou National Nature Reserve, Xizang, China (Araneae, Thomisidae)

**DOI:** 10.3897/BDJ.12.e120347

**Published:** 2024-03-13

**Authors:** Cheng Wang, Jiahui Gan, Xiaoqi Mi

**Affiliations:** 1 Guizhou Provincial Key Laboratory for Biodiversity Conservation and Utilization in the Fanjing Mountain Region, Tongren University, Tongren, China Guizhou Provincial Key Laboratory for Biodiversity Conservation and Utilization in the Fanjing Mountain Region, Tongren University Tongren China

**Keywords:** Crab spider, morphology, new species, southwest China, taxonomy

## Abstract

**Background:**

*Lysiteles* Simon, 1895 contains 64 nominal species distributed in East, South and South Asia. It possesses very high species diversity in China (with 46 and 32 endemics), Bhutan (with 9 and 1 endemic) and Nepal (with 8 and 4 endemics).

In June 2023, a spider survey of Cibagou National Nature Reserve, Xizang, China was carried out. After examination and morphological comparison, two *Lysiteles* species were recognised as new to science.

**New information:**

Two new species of *Lysiteles* Simon, 1895 collected from Cibagou National Nature Reserve, Xizang, China are described: *L.cibagou* sp. nov. (♂♀) and *L.tangi* sp. nov. (♂♀). Diagnostic photos of habitus and copulatory organs and a distributional map are provided.

## Introduction

*Lysiteles* Simon, 1895 is represented by a group of tiny spiders remarkable for having conspicuous blackish-brown markings on the dorsum of carapace and abdomen mostly (Tang et al. 2008). With the series of taxonomic studies conducted, especially those noted on the species from Bhutan and south-western China provided by Prof. Hirotsugu Ono and Dr. Guo Tang, the species number has rapidly increased to 64 (WSC 2024). Amongst the species, most have clear diagnostic illustrations; however, including the genotype, almost 40% have been described from a few specimens of a single sex. Moreover, the genus is poorly defined and could be polyphyletic (Tang et al. 2007), suggesting it needs further taxonomic attention.

The goal of the present work is to describe two new *Lysiteles* species collected from Cibagou National Nature Reserve, Xizang, China.

## Materials and methods

All specimens were preserved in 80%–95% alcohol and are deposited in the Museum of Tongren University (TRU) in Tongren, China. The specimens were examined with an Olympus SZX10 stereomicroscope. After dissection, the vulvae were cleared in trypsin enzyme solution before examination and imaging. Images were taken with a Kuy Nice CCD mounted on an Olympus BX43 compound microscope. Compound focus images were generated using Helicon Focus v. 6.7.1 (Khmelik et al. 2024). All measurements are given in millimetres. Leg measurements are given as total length (femur, patella, tibia, metatarsus, tarsus). References to figures in the cited papers are listed in lowercase type (fig. or figs) and figures in this paper are noted with an initial capital (Fig. or Figs). Abbreviations used in the text and figures are as follows:

**ALE** = anterior lateral eyes; **AME** = anterior median eyes; **CD** = copulatory duct; **E** = embolus; **FD** = fertilisation duct; **MOA** = median ocular area; **PLE** = posterior lateral eyes; **PME** = posterior median eyes; **RTA** = retrolateral tibial apophysis; **S** = spermatheca; **TSP** = transversal sclerotised plate; **VTA** = ventral tibial apophysis.

## Taxon treatments

### Lysiteles cibagou

Wang & Mi sp. nov.

5A34D4D9-91A9-57FE-9900-5833C4C2489A

1BCAF792-9CD3-4F32-B0D2-EA04B25ABEA3

#### Materials

- **Type status:** Holotype. **Occurrence:** individualID: TRU-TD-XZ-001; sex: male; occurrenceID: 861463EB-E43B-5639-A89A-F2C13B346F14; **Taxon:** scientificName: *Lysitelescibagou* sp. nov.; **Location:** country: China; stateProvince: Xizang Autonomous Region; county: Zayu; locality: Cibagou National Nature Reserve; verbatimElevation: 2570 m; verbatimLatitude: 28°41.43′N; verbatimLongitude: 97°2.86′E; **Identification:** identifiedBy: Cheng Wang; **Event:** samplingProtocol: beating shrubs; year: 2023; month: June; day: 26
- **Type status:** Paratype. **Occurrence:** individualID: TRU-TD-XZ-002–008; sex: 2males, 5 females; occurrenceID: E6373605-08CF-5AB2-BC5B-042C5F7F5DCD; **Taxon:** scientificName: *Lysitelescibagou* sp. nov.; **Location:** country: China; stateProvince: Xizang Autonomous Region; county: Zayu; locality: Cibagou National Nature Reserve; verbatimElevation: 2570 m; verbatimLatitude: 28°41.43′N; verbatimLongitude: 97°2.86′E; **Identification:** identifiedBy: Cheng Wang; **Event:** samplingProtocol: beating shrubs; year: 2023; month: June; day: 26

#### Description

**Figure 1.**
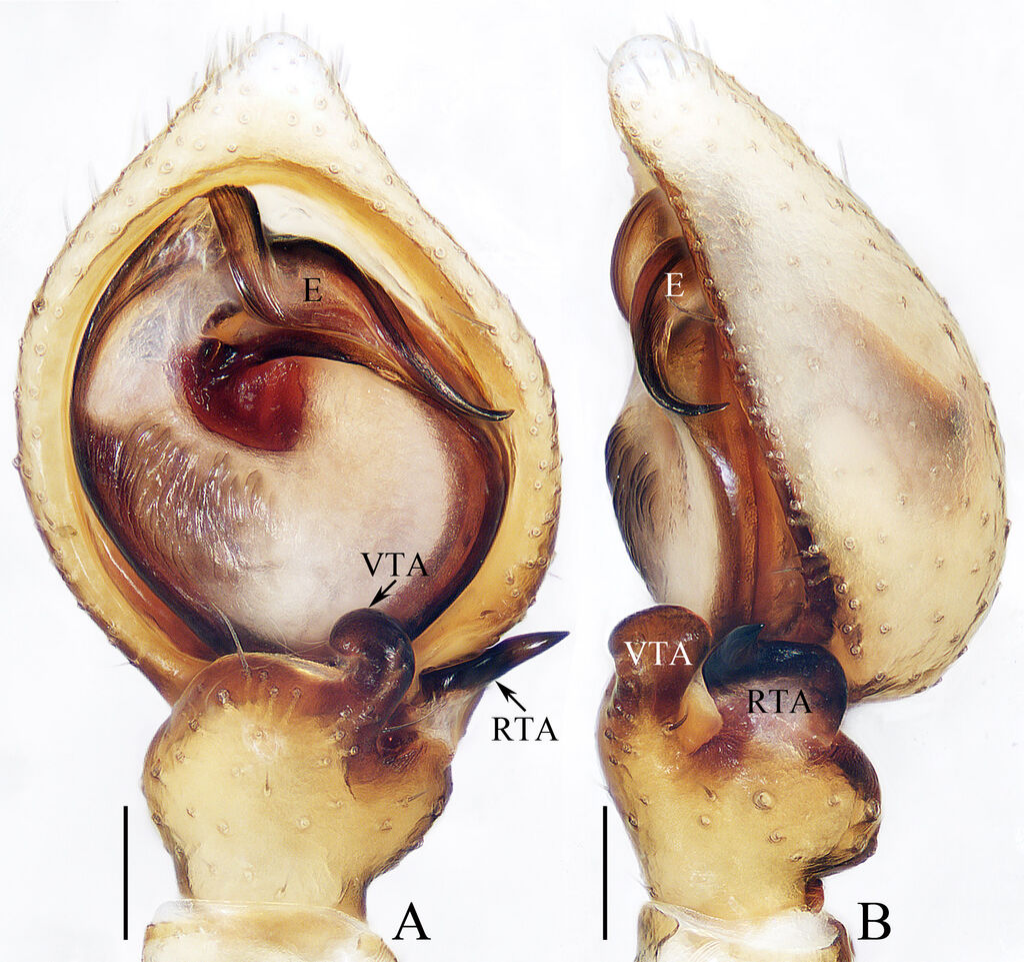
Male palp of *Lysitelescibagou* sp. nov., holotype (TRU-TD-XZ-001). **A** ventral view; **B** retrolateral view. Scale bars = 0.1 mm.

**Figure 2.**
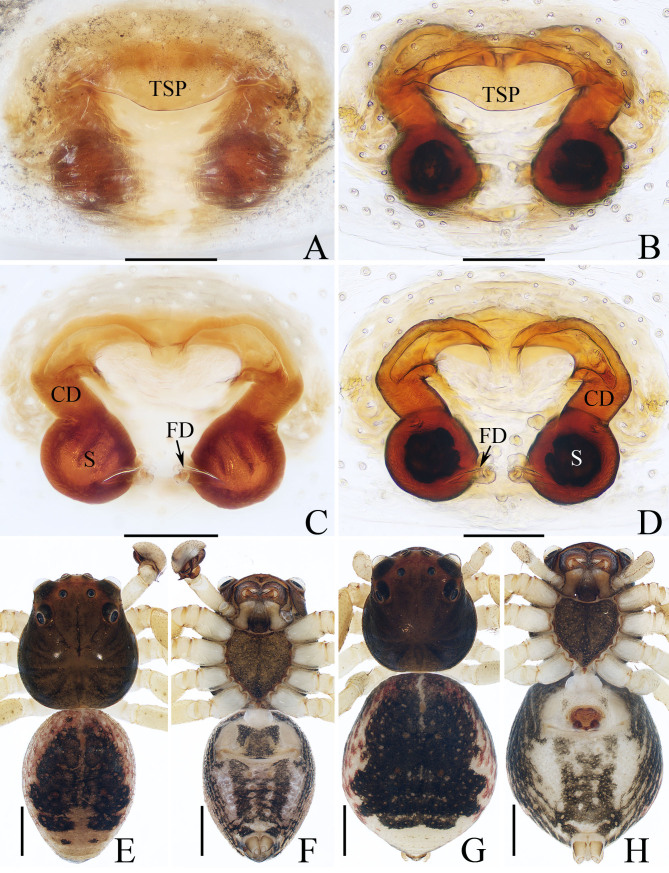
Male holotype (TRU-TD-XZ-001) and female paratype (TRU-TD-XZ-004) of *Lysitelescibagou* sp. nov.; **A, B** epigyne, ventral view; **C, D** vulva, dorsal view; **E** holotype habitus, dorsal view; **F** ditto, ventral view; **G** female paratype habitus, dorsal view; **H** ditto, ventral view. Scale bars = (A–D) 0.1 mm; (E–H) 0.5 mm.

**Male** (holotype, TRU-TD-XZ-001). Total length 2.93. Carapace 1.35 long, 1.23 wide; Abdomen 1.61 long, 1.17 wide. Eye sizes and inter-distances: AME 0.11, ALE 0.21, PME 0.06, PLE 0.16; AME–AME 0.13, AME–ALE 0.11, PME–PME 0.21, PME–PLE 0.25; MOA 0.30 long, front width 0.34, back width 0.38. Measurements of legs: I 5.63 (1.75, 0.55, 1.45, 1.18, 0.70), II 5.66 (1.70, 0.58, 1.48, 1.20, 0.70), III 3.74 (1.18, 0.43, 0.95, 0.73, 0.45), IV 3.90 (1.25, 0.40, 1.00, 0.80, 0.45). Carapace (Fig. 2E) red-brown to black, with three oblique or longitudinal dark stripes posteriorly on cephalon, and pairs of indistinct, lateral dark stripes on thorax. Chelicerae (Fig. 2F) red-brown. Endites (Fig. 2F) pale yellow, with pale, inner-distal areas bearding dense brown setae. Labium (Fig. 2F) brown to dark. Sternum (Fig. 2F) dark brown, almost as long as wide, with re-curved anterior margin and gradually narrowed postero-medially. Legs pale to yellow, with sparse dark spots. Abdomen (Fig. 2E, F) oval, dorsum mahogany to dark, with prominent, median dark marking; venter brown to dark brown, with centre, longitudinal dark band bearing five pairs of dots. Palp (Fig. 1A, B): tibia slightly wider than long; ventral tibial apophysis (VTA) short, prolaterally curved into inverted C-shape; retrolateral tibial apophysis (RTA) broad and flat, acutely narrowed distally into spiny portion; cymbium longer than wide, tapered distally; bulb flat, with sperm duct extending along the submargin; embolus (E) rises at antero-apical portion of bulb, postero-retrolaterally extending, spiralled into ca. two circles, with pointed tip directed dorsally.

**Female** (paratype, TRU-TD-XZ-004). Total length 3.02. Carapace 1.23 long, 1.16 wide; Abdomen 1.81 long, 1.55 wide. Eye sizes and inter-distances: AME 0.12, ALE 0.21, PME 0.06, PLE 0.15; AME–AME 0.14, AME–ALE 0.11, PME–PME 0.23, PME–PLE 0.27; MOA 0.29 long, front width 0.34, back width 0.38. Measurements of legs: I 4.16 (1.28, 0.50, 1.00, 0.80, 0.58), II 4.23 (1.33, 0.50, 1.03, 0.83, 0.55), III 2.94 (0.93, 0.38, 0.70, 0.55, 0.38), IV 3.11 (1.00, 0.38, 0.75, 0.60, 0.38). Habitus (Fig. 2G, H) similar to that of male. Epigyne (Fig. 2A–D): wider than long; with broad, labiate, anterior transversal sclerotised plate (TSP) almost half the epigynal width; copulatory openings beneath the base of transversal sclerotised plate, close to each other; copulatory ducts (CD) thick, curved into arc-shape at anterior half and nearly half the spermathecal diameter in width; spermathecae (S) almost spherical, separated from each other by half their diameter; fertilisation ducts (FD) lamellar, originating from the inner-base of spermathecae.

#### Diagnosis

*Lysitelescibagou* sp. nov. resembles that of *L.conflatus* Tang, Yin, Peng, Ubick & Griswold, 2008 in having similar copulatory organs, especially the postero-retrolaterally extended embolus, broad transversal sclerotised plate and thick copulatory duct, but it can be easily distinguished by the following: 1) the embolus is spiralled (Fig. 1A, B), versus non-spiralled in *L.conflatus* (Tang et al. 2008: fig. 4e, j); 2) the copulatory ducts are curved into an arc-shape at anterior half (Fig. 2B–D), versus curved at proximal and followed by straight, downward extending portions in *L.conflatus* (Tang et al. 2008: fig. 4i, I); 3) the thorax has several pairs of lateral dark stripes (Fig. 2E, G), versus has alternate dark brown and dark yellow stripes in *L.conflatus* (Tang et al. 2008: fig. 4a–c).

#### Etymology

The species name is derived from the name of the type locality; noun in apposition.

#### Distribution

Known only from the type locality in Xizang, China (Fig. 5).

### Lysiteles tangi

Wang & Mi sp. nov.

DBCC2DF2-3EF2-582F-84C2-F02FED3BBED3

02DC55AE-B124-4515-8655-E18C85AA3906

#### Materials

- **Type status:** Holotype. **Occurrence:** individualID: TRU-TD-XZ-009; sex: male; occurrenceID: 34D4A470-E496-54CD-8C0A-D1DEED658CCC; **Taxon:** scientificName: *Lysitelestangi* sp. nov.; **Location:** country: China; stateProvince: Xizang Autonomous Region; county: Zayu; locality: Cibagou National Nature Reserve; verbatimElevation: 2880 m; verbatimLatitude: 28°46.62′N; verbatimLongitude: 97°0.86′E; **Identification:** identifiedBy: Cheng Wang; **Event:** samplingProtocol: beating shrubs; year: 2023; month: June; day: 24
- **Type status:** Holotype. **Occurrence:** individualID: TRU-TD-XZ-010–025; sex: 6males, 10 females; occurrenceID: 56128EA8-3090-547A-B07E-6361FA058159; **Taxon:** scientificName: *Lysitelestangi* sp. nov.; **Location:** country: China; stateProvince: Xizang Autonomous Region; county: Zayu; locality: Cibagou National Nature Reserve; verbatimElevation: 2880 m; verbatimLatitude: 28°46.62′N; verbatimLongitude: 97°0.86′E; **Identification:** identifiedBy: Cheng Wang; **Event:** samplingProtocol: beating shrubs; year: 2023; month: June; day: 24

#### Description

**Figure 3.**
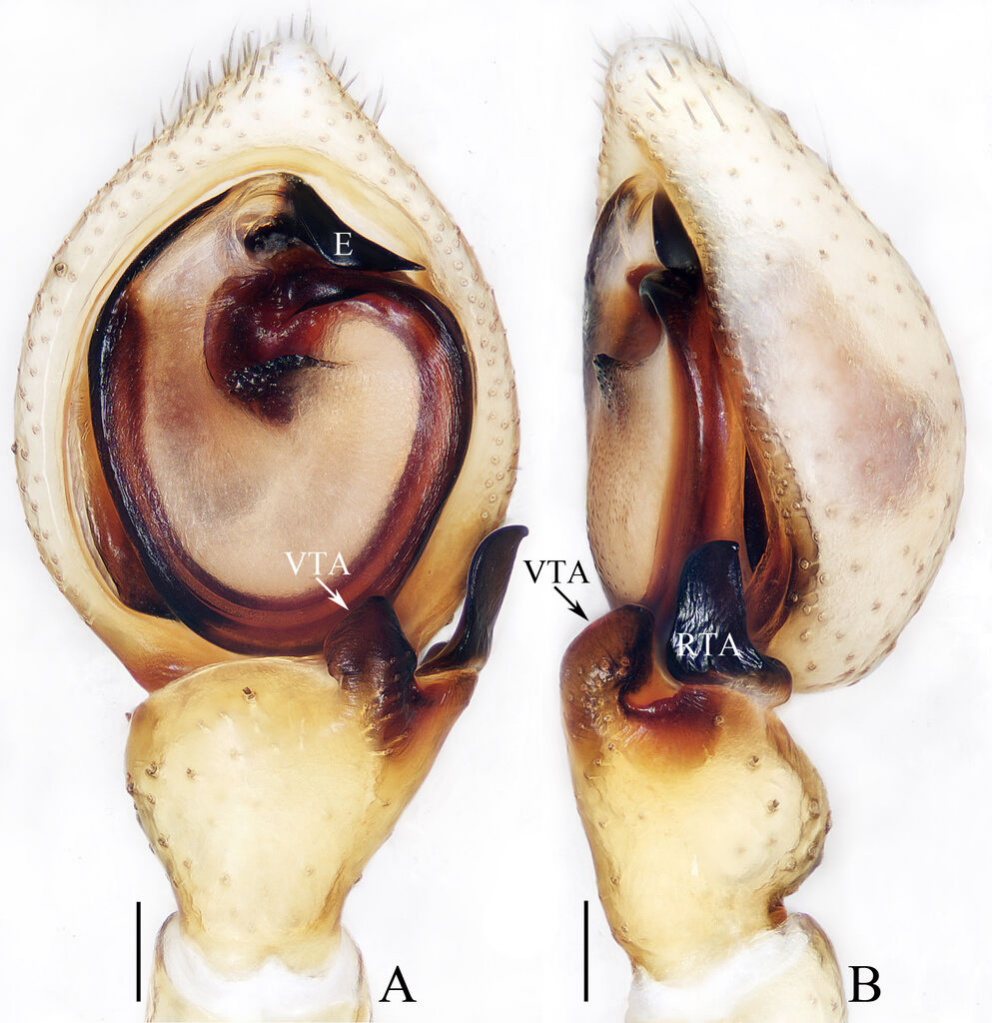
Male palp of *Lysitelestangi* sp. nov., holotype (TRU-TD-XZ-009); **A** ventral view; **B** retrolateral view. Scale bars = 0.1 mm.

**Figure 4.**
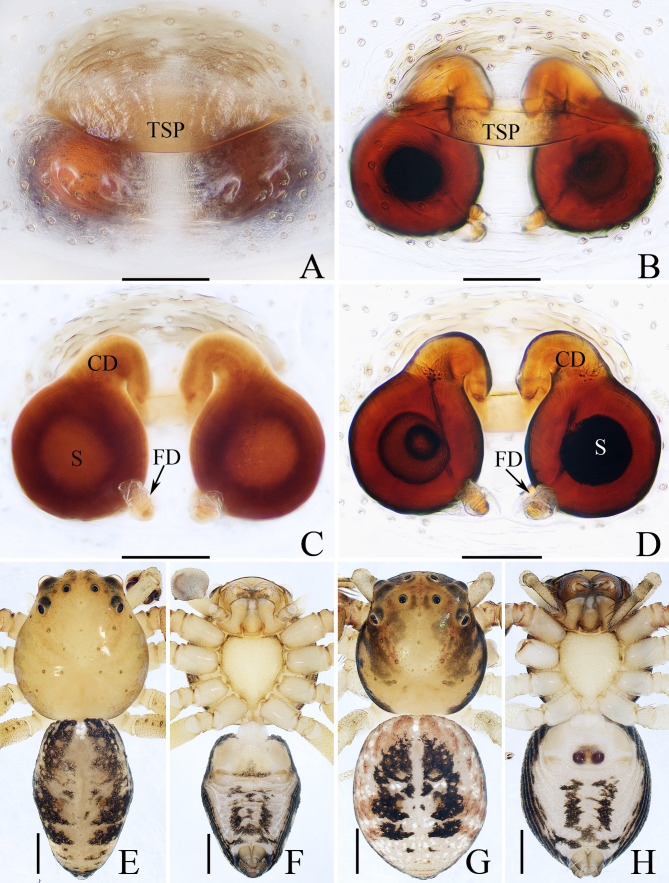
Male holotype (TRU-TD-XZ-009) and female paratype (TRU-TD-XZ-016) of *Lysitelestangi* sp. nov.; **A, B** epigyne, ventral view; **C, D** vulva, dorsal view; **E** holotype habitus, dorsal view; **F** ditto, ventral view; **G** female paratype habitus, dorsal view; **H** ditto, ventral view. Scale bars = (A–D) 0.1 mm; (E–H) 0.5 mm.

**Male** (holotype, TRU-TD-XZ-009). Total length 3.76. Carapace 1.88 long, 1.67 wide; Abdomen 1.91 long, 1.24 wide. Eye sizes and inter-distances: AME 0.13, ALE 0.24, PME 0.08, PLE 0.18; AME–AME 0.16, AME–ALE 0.13, PME–PME 0.28, PME–PLE 0.32; MOA 0.32 long, front width 0.41, back width 0.46. Measurements of legs: I 7.49 (2.20, 0.83, 1.88, 1.63, 0.95), II 7.64 (2.33, 0.78, 1.93, 1.65, 0.95), III 4.98 (1.50, 0.63, 1.25, 1.00, 0.60), IV 5.08 (1.60, 0.55, 1.25, 1.08, 0.60). Carapace (Fig. 4E) pale yellow, with slightly elevated cephalon and sub-oval thorax. Chelicerae, endites and labium pale yellow. Endites (Fig. 4F) longer than wide, bearing dense inner-distal brown setae. Sternum (Fig. 4F) pale yellow, with re-curved anterior margin and gradually narrowed posteromedially. Legs pale to dark yellow, with sparse dark brown spots. Abdomen (Fig. 4E, F) elongated, dorsum pale yellow to dark, with antero-marginal sliver spots, pair of median muscle depressions and prominent dark marking; venter pale to dark, with centre, longitudinal, dark band. Palp (Fig. 3A, B): tibia longer than wide; ventral tibial apophysis (VTA) short, slightly curved inwards medially and blunt apically; retrolateral tibial apophysis (RTA) strongly sclerotised, sheet-shaped, widened at base, slightly curved towards retrolateral side distally; cymbium longer than wide; bulb flat, with sperm duct extending along the sub-margin; embolus (E) strongly sclerotised, originating from antero-apical portion of bulb, retrolaterally extending, slightly curved medially and with pointed end.

**Female** (paratype, TRU-TD-XZ-016). Total length 3.35. Carapace 1.57 long, 1.46 wide; Abdomen 1.81 long, 1.46 wide. Eye sizes and inter-distances: AME 0.14, ALE 0.23, PME 0.08, PLE 0.17; AME–AME 0.17, AME–ALE 0.13, PME–PME 0.29, PME–PLE 0.32; MOA 0.32 long, front width 0.42, back width 0.46. Measurements of legs: I 5.46 (1.63, 0.68, 1.30, 1.10, 0.75), II 5.51 (1.65, 0.63, 1.35, 1.13, 0.75), III 3.80 (1.15, 0.50, 0.95, 0.70, 0.50), IV 3.98 (1.25, 0.50, 0.95, 0.78, 0.50). Habitus (Fig. 4G, H) similar to that of male, except with irregular dark brown markings on carapace. Epigyne (Fig. 4A–D): wider than long, with broad, labiate, anterior transversal sclerotised plate (TSP) almost equal to the epigynal width; copulatory openings beneath the median portion of the base of transversal sclerotised plate; copulatory ducts (CD) short, strongly curved medially; spermathecae (S) almost spherical, separated from each other about half their diameter; fertilisation ducts (FD) originating from the inner-base of spermathecae, lamellar.

#### Diagnosis

*Lysitelestangi* sp. nov. closely resembles that of *L.bhutanus* Ono, 2001, no matter in habitus nor copulatory organs, but it can be distinguished by the following: 1) the retrolateral tibial apophysis is widened at base and longer than the ventral tibial apophysis in retrolateral view (Fig. 3B), versus almost equal in width and almost as long as the ventral tibial apophysis in *L.bhutanus* (Ono 2001: figs 46, 47); 2) the copulatory openings are below the most anterior portions of spermathecae (Fig. 4C, D), versus beyond the most anterior portions of spermathecae in *L.bhutanus* (Ono 2001: fig. 50). The female is also similar to that of *L.linzhiensis* Hu, 2001 in having similar habitus and epigyne, but it can be easily distinguished by the transversal sclerotised plate, which is labiate and almost equal in width to epigyne (Fig. 4A, B), versus sub-triangular and about half the epigynal width in *L.linzhiensis* (Hu 2001: fig. 8-206-2).

#### Etymology

The specific name is a patronym of the late Chinese arachnologist, Dr. Guo Tang, who has significantly contributed to the taxonomy of Chinese *Lysiteles*; noun (name) in genitive case.

#### Distribution

**Figure 5.**
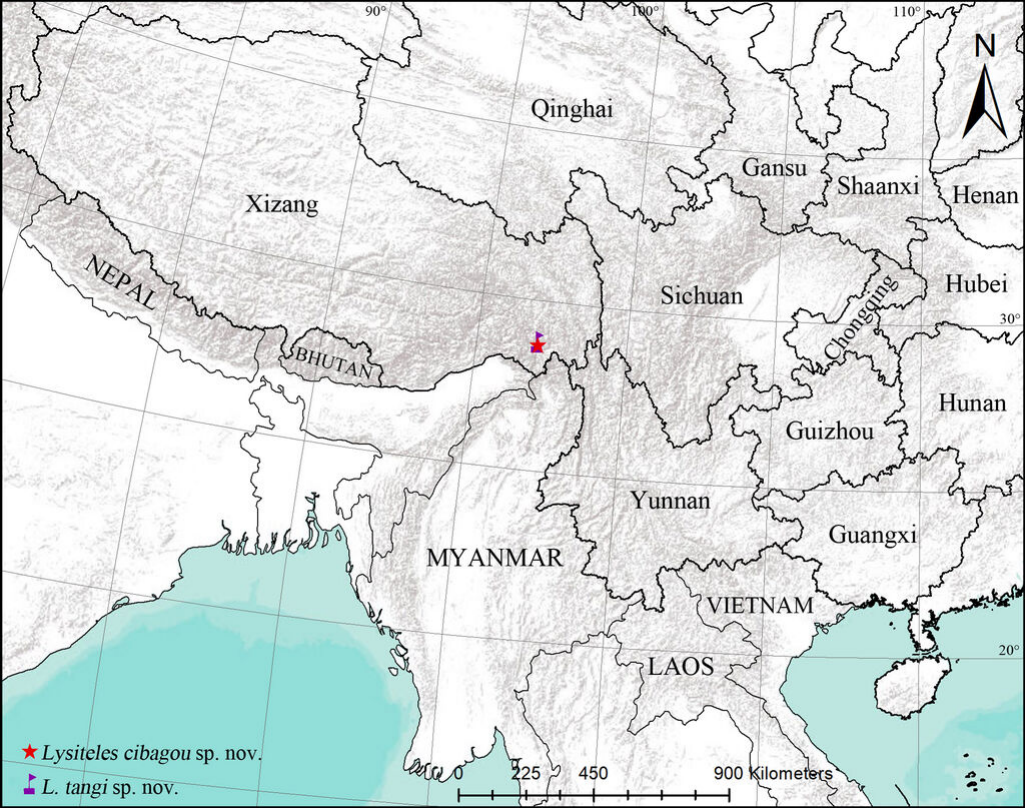
Distributional records of the *Lysiteles* spp.

Known only from the type locality in Xizang, China (Fig. 5).

#### Taxon discussion

The pairing has been supported by the unpublished molecular evidence.

## Supplementary Material

XML Treatment for Lysiteles cibagou

XML Treatment for Lysiteles tangi
